# The Health Care Cost of Dying: A Population-Based Retrospective Cohort Study of the Last Year of Life in Ontario, Canada

**DOI:** 10.1371/journal.pone.0121759

**Published:** 2015-03-26

**Authors:** Peter Tanuseputro, Walter P. Wodchis, Rob Fowler, Peter Walker, Yu Qing Bai, Sue E. Bronskill, Douglas Manuel

**Affiliations:** 1 Bruyère Research Institute, Bruyère Centre of Learning, Research and Innovation in Long-Term Care, Ottawa, Ontario, Canada; 2 Ottawa Hospital Research Institute, Clinical Epidemiology Program, Ottawa, Ontario; 3 Institute for Clinical Evaluative Sciences, Ottawa, Ontario, Canada; 4 Institute for Health Policy, Management & Evaluation, University of Toronto, Ontario, Canada; 5 Department of Medicine and Department of Critical Care Medicine, Sunnybrook Hospital, University of Toronto, Toronto, Ontario, Ontario; 6 Health Analysis Division, Statistics Canada, Ottawa, Ontario, Canada; 7 Department of Family Medicine, University of Ottawa, Ottawa, Ontario, Canada; 8 Toronto Rehabilitation Institute, Toronto, Ontario, Canada; Kuopio University Hospital, FINLAND

## Abstract

**Background:**

Coordinated and appropriate health care across sectors is an ongoing challenge, especially at the end-of-life. Population-level data on end-of-life health care use and cost, however, are seldom reported across a comprehensive array of sectors. Such data will identify the level of care being provided and areas where care can be optimized.

**Methods:**

This retrospective cohort study identified all deaths in Ontario from April 1, 2010 to March 31, 2013. Using population-based health administrative databases, we examined health care use and cost in the last year of life.

**Results:**

Among 264,755 decedents, the average health care cost in the last year of life was $53,661 (Quartile 1-Quartile 3: $19,568-$66,875). The total captured annual cost of $4.7 billion represents approximately 10% of all government-funded health care. Inpatient care, incurred by 75% of decedents, contributed 42.9% of total costs ($30,872 per user). Physician services, medications/devices, laboratories, and emergency rooms combined to less than 20% of total cost. About one-quarter used long-term-care and 60% used home care ($34,381 and $7,347 per user, respectively). Total cost did not vary by sex or neighborhood income quintile, but were less among rural residents. Costs rose sharply in the last 120 days prior to death, predominantly for inpatient care.

**Interpretation:**

This analysis adds new information about the breadth of end-of-life health care, which consumes a large proportion of Ontario’s health care budget. The cost of inpatient care and long-term care are substantial. Introducing interventions that reduce or delay institutional care will likely reduce costs incurred at the end of life.

## Introduction

Health care utilization increases at the end-of-life.[1, 2] The baby boomer cohort effect[3] coupled with an extension of life-expectancy[4] is leading to an increasingly aged population. This has led to great concern among funders and policy makers that elder and end-of-life care will place unprecedented strain on the publicly funded health care system. However, there is little population- and system-wide data to identify the relative contributions of patient characteristics and components of healthcare delivery responsible for healthcare costs at the end of life.[5]

A series of Canadian reports have examined health care use at the end of life in Manitoba[2], British Columbia[6], Saskatchewan[7], and the Atlantic Provinces[8], focusing on specific aspects (e.g., trajectories of death) and specific health sectors. Similar studies have occurred in other jurisdictions.[9–13] In the United States, the Dartmouth Healthcare Atlas has examined variations in end-of-life costs among those with chronic illness who are over 65 years, focusing on hospital, hospice, and physician care.[14, 15] Other studies have examined how various factors such as age, sex, and geographic region affect end-of-life health care expenditures in select populations.[13, 16–20] Very few studies, however, have examined health care use and cost at a population level and across an array of health sectors.[2, 7]

In this study, we systematically examined both use and costs across a comprehensive set of healthcare sectors in Ontario, Canada, a province of over 13 million residents with nearly universal health care coverage.[21] This includes coverage for all residents for costs associated with acute care hospitalizations, physician visits, emergency room visits, long-term care, home care, and complex continuing care. Medications are also insured for those over 65 years and for those receiving social assistance. To our knowledge, no other study has examined end-of-life health care use and cost for such a large population and for such a breadth of health sectors. We aim to inform health care practitioners on the broad health care use of patients prior to death. At a population level, we look beyond the effects of demographic pressures, to examine the relative contributions of sector-specific health care use to overall costs as death approaches. This study will help policy makers and health planners foresee future needs, and highlight areas where care can be optimized.

## Materials and Methods

We conducted a retrospective cohort study examining health care use and cost incurred by decedents in their last year of life. We captured all deaths in a 3-year period, from April 1, 2010 to March 31, 2013 (fiscal year (FY) 2010/11 to 2012/13) in Ontario, Canada. Using encrypted health card numbers as unique identifiers, records of health care use and costs were linked across various administrative databases. This study has been approved by the research ethics board at the Institute for Clinical Evaluative Sciences, at Sunnybrook Health Sciences Centre and at Ottawa Hospital Research Institute. No written consent was obtained; all data were encrypted using health card numbers as unique identifiers. Thus all records used were de-identified and anonymized.

### Data sources and definitions

Deaths were identified using the Ontario Registered Persons Database (RPDB). The databases used to identify health care use are outlined in Table 1. We captured decedents who had at least one health record in the 365 days prior to death. We described the age and sex distribution of these individuals, captured one year prior to death in the RPDB. The decedents’ socioeconomic status was measured using their neighborhood income one-year prior to death. Following well established methods, both neighborhood income and rurality were captured by linking to Statistics Canada census data using postal codes.[22]

**Table 1:** Databases used to record health care use and cost at the end-of-life.

Health care Sector	Database	Description
**Continuing Care**		
Long-term Care	Continuing Care Reporting System (CCRS)	Population-based resident information for over 600 publicly funded residential care homes with 24-hour nursing care
Complex Continuing Care	CCRS	Population-based information for all patients staying in a designated complex continuing care bed. These individuals are typically deemed to be in a non-acute state, but still in need for treatment (e.g., rehabilitation) in an institution
Home Care	Home Care Database (HCD) Resident Assessment Instrument-Home Care (RAI-HC)	Data from the Ontario Association of Community Care Access Centers, responsible for providing all publicly funded home care
Rehabilitation	National Rehabilitation Reporting System (NRS)	Data from participating adult inpatient rehabilitation facilities and programs across Ontario
**Acute Care**		
Inpatient without ICU	CIHI-DAD*	Administrative, clinical, and demographic data on all hospital discharges in Ontario
Inpatient with ICU	CIHI-DAD	Individuals with at least one Intensive Care Unit (ICU) visit in their last year of life
Emergency Department	National Ambulatory Care Reporting System (NACRS)	Captures all emergency department visits in Ontario
**Outpatient Care**		
Outpatient clinics	NACRS	Select outpatient visits held in hospitals, including dialysis clinics and cancer care clinics
Physician Billings	Ontario Health Insurance Plan (OHIP) Claims Database	Claims data for physicians in Ontario—includes claims in both inpatient and outpatient settings.
Non-physician Billings	OHIP	Health professionals for provincially insured services, such as select midwives, oral surgeons, chiropractors, optometrists, and physiotherapists. Some care may occur for inpatients
Laboratory	OHIP	Outpatient laboratory services. Does not include laboratory services for inpatients
Drugs/Devices	Ontario Drug Benefit (ODB), Assistive Devices Program (ADP)	Drugs for those over 65 years, on social assistance, residents of LTC, home care recipients, Trillium drug program and special drugs program recipients for those qualifying for assistance. Select medically-necessary devices including home oxygen and respiratory devices.

*CIHI-DAD: Canadian Institute for Health Information-Discharge Abstract Database

### Statistical analysis

All records of health care use paid for by the provincial Ministry of Health and Long Term Care (MOHLTC) in the last year of life were retrieved. The cost associated with each record was estimated using costing methods developed for health administrative data described elsewhere.[23] Briefly, we’ve taken a payer (MOHLTC) costing perspective, using person-level health care expenditures that accounts for data for health care utilization and cost information per use. Cost information for sectors (e.g., hospitals, complex continuing care, rehab) that have global budgets (e.g., by institution or by health region) are determined using a top-down approach through case-mix methodology. Sectors that have fee payments associated with each use (e.g., drug cost, or cost paid out to physician) have costs estimated directly. All costs were expressed in 2013 Canadian dollars; we inflated past costs using healthcare specific yearly Consumer Price Index reported by Statistics Canada. Health sector cost for the population was the sum of all costs among decedents captured within each respective sector. We also examined total cost within each sector by month prior to death. All statistical tests were two-tailed and p = 0.05 was used to determine statistical significance. We used SAS 9.3 (SAS Institute Inc., Cary, NC) for all analyses.

## Results

### Cohort Characteristics

We observed 264,755 deaths in FY 2010/11 to FY 2012/13. Almost all decedents (98.9%) had at least one health care record in the last year of life, with roughly equal numbers of males and females (Table 2). The majority of deaths (58.1%) was among those 75–94 years, and occurred in urban areas (84.7%—similar to the proportion of all Ontario urban residents).There was a consistent gradient of higher proportions of decedents residing in lower income neighborhoods (p<0.0001).

**Table 2:** Socio-demographic characteristics of health care users in the last year of life, by health care sector, Ontario decedents, Fiscal Year 2010/11 to 2012/13.

	Decedents (n)	%	Average Cost($)	Q1 ($)**	Q3 ($)**
All Decedents	264,755	100%	53,661	19,568	66,875
Decedents with 1+ Use	261,713	98.9%	54,285	20,382	67,256
Male*	127,195	48.6%	54,174	17,572	68,334
Female*	134,518	51.4%	54,390	23,343	66,424
Age, year*					
<19	2,844	1.1%	46,110	1,641	38,725
19–44	7,636	2.9%	47,661	1,530	64,770
45–54	13,629	5.2%	54,744	8,582	73,953
55–64	27,486	10.5%	58,441	14,670	75,534
65–74	42,604	16.3%	59,652	20,179	75,847
75–84	74,272	28.4%	55,918	22,416	69,611
85–94	77,891	29.8%	50,663	24,740	63,009
95+	15,351	5.9%	46,829	28,494	57,537
Neighborhood income*					
Quintile 1	59,382	22.7%	55,316	20,118	68,480
Quintile 2	54,361	20.8%	54,710	20,221	68,176
Quintile 3	50,180	19.2%	53,706	20,773	66,504
Quintile 4	48,859	18.7%	54,439	21,619	67,014
Quintile 5	45,859	17.5%	53,706	21,023	66,389
Rurality*					
Urban	221,736	84.7%	55,456	21,066	68,437
Rural	38,904	14.9%	48,805	18,617	62,040

* Among decedents with 1+ health care use in the last year of life.

** Quartile 1 (Q1) and Quartile 3 (Q3) represent values at the 25^th^ and 75^th^ percentiles of total costs.

### Healthcare Utilization in the Final Year of Life

Average cost in the last year of life was $53,661, leading to a total cost of $14.2 billion over three years, or $4.7 billion annually. Average cost rose slightly over the three fiscal years studied, from $52,498 in 2010, to $54,024 in 2011, and $54,451 in 2012. Median cost was lower at $44,423, reflecting the skew effect of high cost decedents. Average costs were similar for men and women, and $6,651 greater for urban residents (Table 2). Costs were generally lower in the extremes of age, and peaked at an average of $59,652 in the 65–74 year age group.

### Healthcare Costs by Sector

Table 3 shows the breakdown of total cost across all health sectors among all decedents. Inpatient costs (42.9% of all costs) led the way with LTC costs (15.5%), physician costs (10.0%), and home care costs (8.3%) far behind.

**Table 3:** Health care use and cost in the last year of life, by health care sector, Ontario decedents, Fiscal Year 2010/11 to 2012/13.

	**Health Care Users (n)**	**Proportion of all Decedents**	**Average Cost Among Users**	**Q1** **	**Q3** **	**Total Cost in Millions (% of total costs)**
Continuing Care Sectors						
Long-term Care	64,085	24.2%	$34,381	$25,143	$45,336	$2,203 (15.5%)
Home Care	159,616	60.3%	$7,347	$1,609	$9,076	$1,172 (8.3%)
Complex Continuing Care	29,775	11.2%	$30,575	$4,438	$32,902	$910 (6.4%)
Rehabilitation	9,981	3.8%	$23,611	$9,839	$32,324	$235 (1.7%)
Acute Care Sectors						
Inpatient without ICU	141,018	53.3%	$22,830	$7,075	$27,671	$3,219 (22.7%)
Inpatient with1+ ICU*	56,309	21.3%	$51,011	$14,509	$59,573	$2,872 (20.2%)
Emergency Department	219,542	82.9%	$1,535	$699	$1,993	$336 (2.4%)
Outpatient Care Sectors						
Physician Billings	260,332	98.3%	$5,431	$1,979	$7,131	$1,413 (10%)
Outpatient clinics	87,480	33.0%	$10,531	$962	$12,364	$921 (6.5%)
Drugs/Devices	235,670	89.0%	$3,306	$1,027	$4,075	$779 (5.5%)
Non-physician Billings	129,244	48.8%	$659	$50	$1,342	$85 (0.6%)
Laboratory	209,472	79.1%	$272	$100	$358	$56 (0.4%)
All Decedents	264,755	100%	$53,661	$19,568	$66,875	$14,207 (100%)

* Includes all hospitalizations for individuals who had at least 1 Intensive Care Unit (ICU) stay in any of their hospitalization.

** Quartile 1 (Q1) and Quartile (Q3) represent values at the 25^th^ and 75^th^ percentiles of total costs among health care uses in each sector.

Table 3 also highlights the proportion of all decedents with at least one record within each respective health sector, and the associated average sector cost among users. In the final year of life, 24.2% spent at least one day in a long-term care (LTC) facility in the 12 months prior to death, at an average annual cost of $34,381 per resident. In contrast, 60.3% received home care at an average annual cost of $7,347.

A large proportion of decedents in their last year of life had at least one: physician visit (98.3%), drug/device paid for by the provincial insurance (89.0%), an emergency room visit (82.9%), or a laboratory test (79.1%). These services corresponded to relatively small average annual costs.

The average cost for inpatient rehabilitation among users was high, but only a small proportion (3.8%) of all decedents used this service. The average cost for admissions to hospital (74.5% of all decedents) was $30,872. The average hospital cost among those with at least one ICU visit (21.3% of decedents) in the last year of life was highest among all sectors at $51,011. The median for all sectors, with the exception of long-term care, was lower than the mean, again reflecting the skew effect of high cost users.

### Healthcare Costs by Sector as Death Approaches

**Fig 1:**
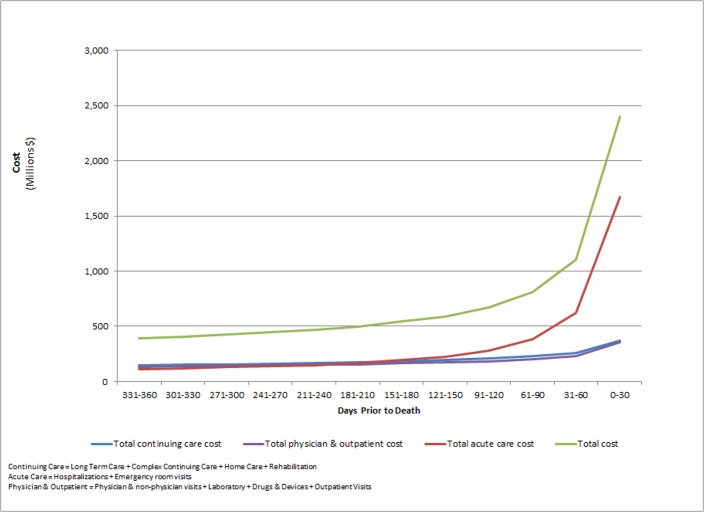
Health care cost in 30-day increments prior to death, by groups of health care sectors, Ontario decedents, Fiscal Year 2010/11 to 2012/13.

Fig. 1 shows total cost leading up to death, divided by 30 day periods. The total population cost associated with acute care, outpatient care, and continuing care consistently increased closer to death. Acute care costs, however, diverged by increasing rapidly in the last 120 days—dominated by rising inpatient costs (95% of acute care costs). Conversely, outpatient and continuing care were relatively stable until the last 30 days, where costs rose by 63% and 33%, respectively (c.f. 181% for acute care in the same period).

Total population cost in the final 30 days was more than 6 times larger than in the first 30 days of the last year of life; for inpatient costs, there was more than 15 fold increase. Complex continuing care, emergency room visits, and physician costs increased between 5–10 fold. Home care and rehabilitation costs increase more than 3 fold and the remaining sectors increased less than 1.5 fold.

## Discussion

In this study of healthcare utilization and costs among all decedents in Ontario, we found that the cost of health care in the last year of life is high. Decedents, who constituted 0.67% of the population, consumed $4.7 billion dollars annually, or approximately 10% of Ontario’s total health care budget.[24] Compared to an age-sex matched survivor cohort during the same period, decedents consumed 6.7 times greater mean cost. End-of-life cost is driven largely by an increase in inpatient hospital costs in the last 120 days of life. If inpatient costs in this period increased proportionately to all other sectors (86% increase instead of a 502% increase), $2.3b of total cost would be reduced. Some hospitalizations and other health care use near the end-of-life may be appropriate (e.g., for curative efforts when death is not imminent or for when symptom relief cannot be achieved otherwise). Regardless, some hospitalizations at the end of life can be potentially preventable.[25–27] In our study, only 9.1% of hospitalizations in the last year of life were admitted with palliative care as the main patient service or the main responsible diagnosis, accounting for 7.3% of total inpatient cost (data not shown). In Ontario, similar to other Canadian and international jurisdictions, there is a push to care for the aging outside of the hospital setting.[28–32]

While over three-quarters of decedents separately used physician services, medications and devices, outpatient laboratory services, and emergency rooms in the last year of life, these services combined contributed less than 20% to total costs. This proportion is lower than other periods of life (26.6% of Ontario’s health care budget is spent on physician services alone).[24] It is apparent that to achieve potentially large cost savings at the end of life, efforts should focus on reducing hospitalizations. Such an approach will likely simultaneously improve the dying experience since most people prefer to spend their last days of life at home.[33–35]

Furthermore, about one in four people at the end-of-life spend time in a LTC home, at a substantial cost, while sixty percent received home care at a much smaller cost (Table 3). It seems prudent, from a direct cost perspective, to identify and target the modifiable predictors of LTC entry so that people may stay at home longer with supports. While those in LTC are generally in higher need, a series of *Balance of Care* reports in Ontario have shown that there are LTC residents who may be reasonably cared for at home at lower cost.[36] Debates and efforts to care for patients in optimal settings are globally pervasive.[30–32]

This analysis adds new information about the breadth of health care costs in the last year of life. Very few previous studies have estimated population-level end of life costs[7, 37], and most have been studied in the Canadian context. Our mean cost of more than $50,000 per decedent is higher than previous population-based estimates; studies from British Columbia (2004–06)[16], Saskatchewan (2003–04)[7], and Manitoba (2000–01)[2] estimated averages of $20,705, $31,492, and a median of less than $15,000, respectively. Our estimate was likely higher due to inclusion of several health sectors not included in other studies, and from temporal escalation of costs. For example, the BC study examined only hospital, ambulatory, and prescriptions drug costs, while Saskatchewan added LTC and home care but did not include outpatient clinics, laboratory services, non-physician services, and rehabilitation costs. Other cost estimates have focused on disease specific end-of-life cohorts, and on specific segments (e.g. older adults) of the population.[14, 15, 38–40] Many of these originate from the United States, often limited to those over 65 years of age and do not take into account nursing home costs[37]. Population inclusions varied considerably; cost estimates accordingly range widely from about $40,000 for Medicare and Medicaid patients[41, 42] to $95,776 for people with multiple cardiovascular disease risk factors.[43]

Average cost peaked for those dying at 65–74 years and declined over 75 years. This pattern is similar to a 2008 study[7], but contrary to older Canadian studies that found increasing cost over 75 years.[2, 44] This discrepancy is potentially explained by healthier populations at older ages (healthy aging hypothesis). We did not find significant differences in end-of-life cost across income quintiles, in contrast with previous Canadian studies.[2, 16] We also found higher care cost for urban residents prior to death, while previous studies have reported mixed results [2, 7, 17]. There are many potential reasons for higher urban costs, including improved access to care, and differences in patient preferences with respect to places of care near the end of life.[45]

### Strengths

We examined a wide array of health care services at the end of life for a large, population-based decedent cohort. This is possible in Ontario, comprising of approximately 40% of the Canadian population, where well-developed health administrative databases are linked at an individual level for a range of publically-funded health services. Costing was estimated accordingly at an individual record level, using detailed costing methodology taking into account the intensity and duration of care.[23] Previous studies have focused on specific populations (e.g. seniors, disease-groups, insurance subscribers) and/or on a select set of health care sectors, and included smaller sample sizes.

### Limitations

Our study is descriptive in nature, and does not directly address quality of care; nevertheless, it can be inferred that a proportion of health care utilization near the end of life, such as certain emergency room visits, hospital admissions and medications, are potentially preventable and burdensome for the patient. We use administrative databases to capture health care use and direct costs in the last year of life. Each database has limitations in population coverage. For example, the OHIP database, although capturing the vast majority of all physician services, does not include some services provided in provincial psychiatric hospitals and services provided by physicians on an alternate funding plan. The Ontario Drug Benefit program, as another example, provides coverage only to those over 65 years of age and those on social assistance. Our perspective was from the public health care system. Due to lack of data, we did not include the cost of publically subsidized hospices in Ontario. Approximately 2,500 Ontarians die in such hospices annually. Overall, it is thus likely that our estimation that 10% of the health care budget is due to end-of-life costs is an underestimation of the true proportion.

Furthermore, not included are health care use and costs paid out-of-pocket.[46] This includes private assisted-living arrangements, private home care support services, and drugs, devices, and non-physician services not covered by the provincial health insurance. We also did not include the substantial indirect costs that are associated with the dying process, including the costs incurred by informal caregivers.[47] Finally, our study was conducted in a Canadian province with extensive universal coverage; our results may not be generalizable to populations with vastly different health care systems.

## Conclusions

This study improves our population-level understanding of health care use at the end-of-life. Knowing, for example, the proportion of the population that used long-term care or home care, or the proportion that were admitted into hospital and/or ICU informs conversations around the end-of-life experience. For policy makers and health care planners, this study identifies the sectors that are important to target from a cost perspective. It has been proposed that future costs in an aging society may be more dependent on policy action rather than inevitable demographic trends.[48] Previous research has shown that higher intensity of care at the end of life doesn’t necessarily lead to better outcomes.[49, 50] Future research is needed to elucidate which care is appropriate, unnecessary, or absent, especially in the last 4 months of life where we’ve shown rising acute care costs. Future research is also needed to elucidate the modifiable, pre-disposing factors that lead to increased health care need, and the interventions that reduce need and allow optimized care to be provided in suitable settings.
